# Cardiac abnormalities in patients with two different mitochondrial myopathy syndromes as detected by cardiovascular magnetic resonance imaging

**DOI:** 10.1186/1532-429X-17-S1-Q83

**Published:** 2015-02-03

**Authors:** Anca R Florian, Anna Ludwig, Sabine Rösch, Udo Sechtem, Ali Yilmaz

**Affiliations:** 1 Cardiology, Uniklinikum Muenster, Muenster, Germany; 2 Cardiology, Robert Bosch Hospital, Stuttgart, Germany

## Background

Chronic progressive external ophtalmoplegia (CPEO) and Kearns-Sayre syndrome (KSS) belong to the broad heterogeneous group of mitochondrial myopathies (MM) - a group of neuromuscular disorders resulting from primary dysfunction of the mitochondrial chain with impaired cellular energy metabolism. Recently, a potentially characteristic myocardial fibrosis pattern with intramural late gadolinium enhancement (LGE) in the left ventricular (LV) inferolateral wall was documented by cardiovascular magnetic resonance imaging (CMR) in these patients.

The present study aimed at characterizing the presence and pattern of cardiac abnormalities in a larger CPEO/KSS patient population by means of CMR. Based on the fact that patients with other MM syndromes may show a concentric LV remodelling/hypertrophy (e.g. **m**itochondrial **e**ncephalopathy with **l**actic **a**cidosis and **s**troke-like episodes - MELAS) this aspect was also evaluated.

## Methods

In a prospective study, 33 CPEO/KSS patients (52±14yrs, 36% male) underwent neurological and cardiac evaluations including CMR studies comprising cine- and LGE-CMR (1.5-Tesla). Additionally, CMR scans were performed in 25 age-, gender- and cardiac risk factor-matched asymptomatic CONTROLS (47±12yrs, 48% male) without known heart disease.

## Results

In the CPEO/KSS group, 18 (55%) patients had at least one pathological finding based on the CMR results: 8 (24%) patients demonstrated an impaired LV-EF (<60%), 3 (9%) patients showed presence of LV hypertrophy and 10 (30%) patients showed non-ischemic LGE with a predominantly intramural pattern, mostly confined to the basal LV inferolateral wall.

There were no significant differences in LV volumes, mass or ejection fraction between CPEO/KSS and CONTROLS. However, CPEO/KSS patients demonstrated significantly higher LV mass/ end-diastolic volume ratios as surrogate for LV concentric remodelling when compared to CONTROLS (0.84±0.24 vs. 0.67±0.11 g/mL, p=0.001).

## Conclusions

CPEO/KSS patients frequently show myocardial fibrosis with a predominantly intramural pattern located in the LV inferolateral wall as depicted by LGE-CMR. Moreover, although overt LV hypertrophy is relatively rare, when compared to matched controls, CPEO/KSS seem to show a concentric LV remodelling already described in other MM syndromes.

## Funding

None.

